# Phenotype & genotype of congenital adrenal hyperplasia due to mutation in the type ii 3β-hydroxysteroid dehydrogenase gene: a report of two Vietnamese families

**DOI:** 10.1186/1687-9856-2015-S1-P50

**Published:** 2015-04-28

**Authors:** Vu Chi Dung, Bui Phuong Thao, Nguyen Ngoc Khanh, Can Thi Bich Ngoc, Nguyen Phu Dat, Nguyen Thi Hoan, Yves Morel

**Affiliations:** 1 Department of Endocrinology, Metabolism and Genetics; National Hospital of Pediatrics, Hanoi, Vietnam; 2 Service d’Endocrinologie Moléculaire et Maladies Rares, Centre de Biologie et Pathologie Est, Bron. France

## 

Congenital adrenal hyperplasia (CAH) is one of the most common inherited metabolic disorders. It includes a group of autosomal recessive disorders caused by the deficiency of one of the enzymes involed in one of the various steps of adrenal steroid synthesis. 3β-Hydroxysteroid dehydrogenase (3β-HSD) deficiency is a rare cause of CAH caused by inactivating mutations in the HSD3B2 gene. Most mutations are located within domains regarded crucial for enzyme function. Our aim is to describe phenotype and to identify mutations of HSD3B2 in two classic β-HSD deficient patients belonging to two apparently unrelated pedigrees. This is a case series study. Family history and clinical manifestations were described. Genomic DNA from these patients was extracted using standard procedures from the peripheral blood leukocytes. Mutation analysis of HSD3B2 was performed using Polymerase chain reaction (PCR) and DNA direct sequencing. Vietnamese 46,XY newborn referred at 2,5th month of life with salt loss associated with hyponatremia (123 nmol/L) and hyperpigmentation. The testes were palpable in the scrotum but associated with a severe hypospadias (micropenis 0.5 cm; posterior). At 4 months of age, a second adrenal crisis has occurred with hyponatremia 127 nmol/L and increased 17OH-Progesterone (26,8 ng/ml) in this 46, XY DSD. This clinical and biological data associated with a sibling with female phenotype deceased at 18 months old after adrenal crisis (1^st^ occurred at 7 days of life) suggest the diagnosis of 3β-HSD deficiency. The sequencing of HSD3B2 confirms the diagnosis because he is homozygous for a missense mutation, pAla161Pro. This mutation affects an aminoacid conserved in all species and is located in one two ahelix involved in the dimerization of the two sub-units of the enzyme. The changing from Alanine to Proline could break the alpha-helix. The same mutation has been found in the other Vietnamese family. The 46,XY newborn referred at 3th month of life with severe dehydration associated with hyponatremia (93 nmol/L) and hyperpigmentation. The testes were palpable in the scrotum but associated with a severe hypospadias (micropenis 0.5 cm; posterior). Clinical presentation and increased 17OH-Progesterone (9.7 ng/ml) in this 46, XY DSD suggest the diagnosis of 3β-HSD deficiency. The sequencing of HSD3B2 also confirms the diagnosis because he is homozygous for a missense mutation, pAla161Pro. The severity of this mutation correlates well with the phenotype in these patients. Parents of two unrelated pedigrees are not consanguinity. This study contributes to a better understanding of the molecular defects of 3β-HSD and of the phenotypic heterogeneity of CAH related to 3β-HSD deficiency.

Written informed consent was obtained from the patient for publication of this Case report (and any accompanying images). A copy of the written consent is available for review by the Editor-in-Chief of this journal.

